# Survival Analysis and Immune Differences of HIV Long-Term Non-progressors in Xinjiang China: A 12-Year Prospective Cohort Observation

**DOI:** 10.1007/s10461-024-04396-x

**Published:** 2024-06-13

**Authors:** Yuefei Li, Yongkang Ni, Qian He, Xiaoyuan Hu, Yu Zhang, Xiaoyan He, Mingjian Ni

**Affiliations:** 1 Public Health School, Xinjiang Medical University, Urumqi, 830011 Xinjiang China; 2 STD/HIV Prevention and Control Center, Xinjiang Uighur Autonomous Region Center for Disease Control and Prevention, Urumqi, 830002 Xinjiang China; 3 HIV/STD Prevention and Control, Yining City Center for Disease Control and Prevention, Ili Kazakh Autonomous Prefecture, 835000 Xinjiang China; 4 Laboratory, Yining Second People’s Hospital (Jinling Sunshine Hospital), Ili Kazakh Autonomous Prefecture, 835000 Xinjiang China

**Keywords:** LTNPs, ART, Survival analysis, Immune difference, LTNPs, TAR, Análisis de supervivencia, Diferencia inmunológica

## Abstract

This 12-year cohort study of 80 long-term non-progressors (LTNPs) observed a cumulative follow-up duration of 628.5 person-years. Among them, 60 received antiretroviral therapy (ART) for a total of 418.6 person-years. Twenty-four deaths occurred during the follow-up period, with an average age of 42.36 years and a lowest 8-year survival rate of 0.90. Cox model analysis revealed that the risk of AIDS-related death was 1.47 times higher for non-marital, non-commercial heterosexual transmission than for injection drug use. Treatment initiation at ages 31–40 was correlated with an elevated risk of mortality, while treatment for 3–10 years reduced mortality risks in untreated LTNPs. Flow cytometry observed significant differences in the proportion of NK cells. Long-term ART (> 2 years) before LTNPs developed AIDS symptoms could lower mortality risk and potentially extend lifespan, especially when it was initiated at a younger age without affecting NK cell balance. Epidemiological and immunological studies on ART-treated LTNPs are vital for advancing HIV treatment and achieving functional cures for AIDS individuals.

**Supplementary Information:**

The online version contains supplementary material available at 10.1007/s10461-024-04396-x.

## Resumen

Este estudio de cohorte de 12 años con 80 no progresores a largo plazo (LTNPs) observó un total acumulado de 628.5 personas-año. De ellos, 60 recibieron terapia antirretroviral (TAR) durante un total de 418.6 personas-año. Se produjeron veinticuatro muertes durante el período del estudio, con una edad promedio de 42.36 años y una tasa de supervivencia más baja de 0.90 a los 8 años. El análisis del modelo de Cox identificó que la transmisión heterosexual no marital ni comercial presentaba un riesgo 1.47 veces mayor de muerte relacionada con el SIDA en comparación con el uso de drogas inyectables. Comenzar el tratamiento entre los 31-40 años mostró incrementos en los riesgos de mortalidad, mientras que 3-10 años de tratamiento redujeron los riesgos de mortalidad en LTNPs no tratados. Se observaron diferencias significativas en las proporciones de células NK desde el punto de vista inmunológico. La TAR a largo plazo (> 2 años) antes de la aparición de síntomas del SIDA en LTNPs podría disminuir el riesgo de mortalidad y potencialmente prolongar la vida, especialmente si se inicia a una edad más temprana sin afectar el equilibrio de las células NK. Los estudios epidemiológicos e inmunológicos sobre LTNPs tratados con TAR son fundamentales para el progreso del tratamiento del VIH y la cura funcional del SIDA.

## Introduction

Long-term non-progressors (LTNPs), a group of HIV-positive individuals, have been identified in the absence of sustained antiretroviral therapy (ART). Known as They remain asymptomatic and naturally control HIV replication over a follow-up period of 7 to 10 years [1, 2]. Their peripheral blood CD4 + T-cell counts remain above 500 cells/µL, and viral loads (VL) stay typically below 10,000 copies/mL. It is estimated that LTNPs make up approximately 1–5% of all people living with HIV (PLWH) [3]. Mimicking their immune response patterns through immunotherapy may eventually lead to a functional cure for HIV [4]. Therefore, describing the physical factors involved in LTNPs is an essential way to explore functional cures for HIV infection. Although ART does not cure the disease, it effectively controls the viral infection and modifies the adaptive immune function of HIV-infected individuals. For example, in elite controllers (EC), ART decreases activation indices but does not appear to increase circulating CD4 T cells [5]. In recent years, both ART and LTNPs have been studied at home and abroad, but there are few studies on ART in LTNPs.

In 2006, Xinjiang initiated free ART for HIV/AIDS individuals. Since 2016, Xinjiang has adopted a comprehensive strategy of “Treating as Much as Possible” for HIV/AIDS, aiming to extend the therapy to all eligible infected individuals. As national policies undergo continuous refinement, some LTNPs have initiated ART. This has led to inquiries into the potential impact of ART on the life expectancy of LTNPs. The efficacy of ART for LTNPs remains a topic of discussion within the international research community.

To address these questions and assess the survival of LTNPs after ART, this study, based on a 12-year prospective cohort of LTNPs in Xinjiang, aims to analyze the survival of LTNPs undergoing treatment, identify influencing factors, and explore immunological differences between this population and other HIV-infected individuals, shedding light on potential factors for non-progression. Overall, the study seeks to provide further support for research on HIV functional cure.

## Study Subjects and Methods

### Patients and Data Collection

**Fig. 1:**
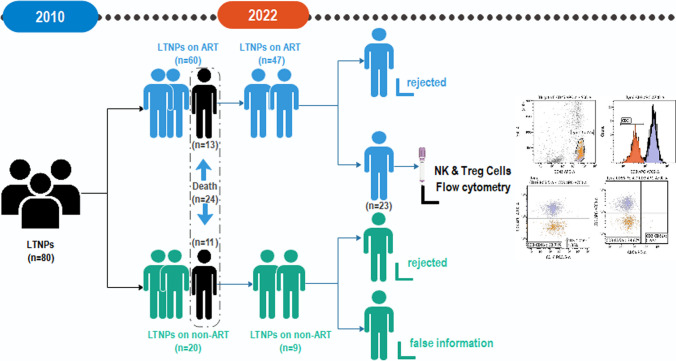
Flow chart of cohort study

Inclusion criteria covered the following aspects: Confirmed positive for HIV antibodies by Western Blot before October 1, 2003; no AIDS-related symptoms confirmed on October 2010; annual CD4 + T-cell counts > 500 cells/µL up to the time of recruitment; age ≥ 15 years; no other HIV opportunistic or infectious diseases (such as tuberculosis, hepatitis B, hepatitis C, Candida albicans, etc.); permanent residents of Yining City; providing written informed consent to participate in the study. Individuals were excluded for the following reasons: receiving antiviral treatment during the period from infection to recruitment; absence of informed consent; communication barriers due to language or other issues; currently pregnant; presence of hepatitis B or C or any signs of acute infection detectable by Polymerase Chain Reaction (PCR). Ultimately, 80 LTNPs from Yining City were included in the study. The cohort study is shown in the flow chart, as shown in Fig. 1.

### Cohort Study Methods

The cohort observation began in October 2010. After inclusion, participants provided their basic information and underwent blood tests to measure CD4 + T-cell counts (as baseline CD4 + T-cell values). The end point of the study was the date of last contact (the date of death for those who died, and the date of last follow-up or lost to follow-up for survivors and those lost to follow-up). The last follow-up date was July 25, 2022. Data censored in the study included refusal to follow-up, relocation, severe illness, and provision of false information.

#### Baseline Data

Baseline information of LTNPs was collected and verified through the Yining City Disease Prevention and Control Center for HIV-infected individuals, including (1) Demographic features: name, age, gender, home address, and transmission route; (2) Disease-related information. Early records were mainly handwritten. To ensure the accuracy of disease-related information, baseline data were documented at cohort inclusion, and laboratory testing data were used as the foundational dataset.

#### Follow-up

The cohort was followed up by locally trained AIDS specialists, including updating information and collecting blood samples. CD4+ T-cell counts and VL tests were performed by designated infectious disease units. Telephone follow-up was performed every six months and face-to-face follow-up was performed annually.

#### Survival Analysis Determinants and Criteria

(1) Death is defined as heart and lung failure in HIV/AIDS individuals. Deaths due to ‘suicide’, ‘accidental death’, and other non-disease-related causes (such as drugs, fighting, injuries, etc.) were excluded. (2) Time of death was reported by family members or discovered during follow-up based on medical death certificates or official registration by law enforcement. (3) Age at infection was defined as the age at which HIV was first confirmed positive according to the local disease prevention and control center’s registration. (4) Baseline CD4+ T-cell counts were tested at cohort inclusion in October 2010. (5) Survival time was defined as the duration from the first confirmed HIV-positive registration to the latest follow-up as of July 25, 2022, calculated in person-years. (6) Censoring/Truncation: Deaths due to causes other than AIDS and related diseases during the follow-up period, as well as loss to follow-up were censored.

#### Missing Value Handling

For patients who relocated or withdrew from the cohort due to serious illness during the follow-up process, their data were truncated. If an individual refused follow-up once or provided false information but subsequently continued to complete the follow-up, their missing survival status was supplemented as “0”. For missing laboratory test values, the mean imputation method was used based on the individual's previous test results.

### Immunological Research Methods

*Control Group Selection* A total of 23 LTNPs on ART who agreed to participate in immunological research and underwent blood sampling were selected from the cohort as the case group. Actively-treated non-progressing HIV-infected individuals (ART) matched on age, gender, and treatment duration on a 1:1 ratio were selected as the control group. Additionally, a matched group of HIV-infected individuals (PLWH) who never received ART on a 1:1 ratio were selected as the blank control.

*Sample Collection* Peripheral venous blood samples (6 mL) from all study subjects were collected on a specific day into EDTA anticoagulant tubes and transported to the laboratory within 24 h. CD4 + T cells, VL, NK cells, and Treg cells were tested using a flow cytometer (Beckman Coulter, DXflex).

### Statistical Methodology

Data processing and statistical analysis were conducted using SPSS 21.0. Categorical variables were analyzed using frequency distributions and I^2^ tests. Survival rates were estimated and survival curves were plotted using the Kaplan–Meier method, and differences in survival rates among different groups were analyzed using the log-rank test. Both univariate and multivariate Cox proportional hazards models were employed to analyze the factors influencing mortality, with AIDS-related death (AIDS-related death = 1, censored event = 0) as the dependent variable. The variables were selected based on univariate analysis with forced inclusion of variables highly relevant to the field, using a criterion of P ≤ 0.1 to establish the Cox proportional hazards model. A significance level was set at P = 0.05 for two-sided tests.

## Results

### Basic Characteristics of LTNPs from 2010 to 2022

A total of 80 LTNPs were included in the study, with a cumulative follow-up duration of 628.5 person-years and an average follow-up duration of 7.9 person-years. As of July 25, 2022, among the 80 LTNPs, 60 had sequentially received ART, with a total treatment duration of 418.6 person-years and an average treatment duration of 7.0 person-years (Table S1). The overall mortality was 24, with an average age at death of 42.36 years (Table 1). Four treatment regimens were employed, including: (1) Kaletra (lopinavir/ritonavir) + lamivudine (3TC) + zidovudine (AZT), (2) efavirenz (EFV) + lamivudine + zidovudine, (3) nevirapine (NVP) + lamivudine + tenofovir disoproxil fumarate (TDF), and (4) efavirenz + lamivudine + tenofovir disoproxil fumarate. Log-rank tests revealed statistical differences in survival time among groups when comparing age at the start of treatment (*χ*^*2*^ = 9.545, *P* = 0.049) and ART duration (*χ*^*2*^ = 43.26, *P* < 0.001).

**Table 1:** Basic characteristics of LTNPs from 2010 to 2022

Variable	LTNP (n = 80)	LTNP on ART (n = 60)	Death (n = 24)	*χ*^*2*^	P value*
Age /years^c^	47.59 ± 4.627	47.0 ± 4.329	47.96 ± 5.069		
Gender^a^					
Male	76 (95.00)	57 (95.00)	24 (100.00)	0.173	0.677
Female	4 (5.00)	3 (5.00)	0 (0.00)		
Age at HIV diagnosis /year^a^					
≤ 30	60 (75.00)	47 (78.33)	19 (79.17)	1.264	0.261
> 30	20 (25.00)	13 (21.67)	5 (20.83)		
Transmission route^a^					
IDU	52 (65.00)	39 (65.00)	17 (70.83)	4.091	0.252
Spouse/fixed sex with positive	11 (13.75)	9 (15.25)	5 (20.83)		
Non-marital, non-commercial heterosexual contact	14 (17.50)	9 (15.25)	2 (8.33)		
Other	3 (3.75)	3 (5.00)	0 (0.00)		
Baseline CD4 + T count^a,b^					
≤ 200	4 (5.00)	4 (6.67)	0 (0.00)	1.925	0.588
201–350	25 (31.25)	19 (31.67)	9 (37.50)		
351–500	16 (20.00)	12 (20.00)	5 (20.83)		
> 500	35 (43.75)	25 (41.67)	10 (41.67)		
Antiviral treatment^a^					
Yes	60 (75.00)	60 (100.00)	16 (66.67)	1.663	0.197
No	20 (25.00)	0 (0.00)	8 (33.33)		
Infection to ART interval/year^a^					
≤ 10	25 (31.25)	25 (41.67)	5 (20.83)	2.518	0.472
11 ~ 15	29 (36.25)	29 (48.33)	10 (41.67)		
16 ~ 20	5 (6.25)	5 (8.33)	0 (0.00)		
> 20	1 (1.25)	1 (1.67)	0 (0.00)		
Age at time of start ART/year^a^					
≤ 30	4 (5.00)	4 (6.67)	1 (4.17)	9.545	0.049
31 ~ 40	38 (47.50)	38 (63.33)	10 (41.67)		
41 ~ 50	17 (21.25)	17 (28.33)	3 (12.50)		
> 50	1 (1.25)	1 (1.67)	1 (4.17)		
CD4 + T cells at the start of ART^a,b^					
≤ 200	9 (11.25)	9 (15.00)	5 (20.83)	3.255	0.516
201–350	19 (23.75)	19 (31.67)	4 (16.67)		
351–500	10 (12.50)	10 (16.67)	2 (8.33)		
> 500	22 (27.50)	22 (36.67)	4 (16.67)		
Duration of ART/year^a^					
≤ 2	4 (5.00)	3 (5.00)	0 (0.00)	43.258	< 0.001
3 ~ 5	13 (16.25)	9 (15.00)	5 (20.83)		
6 ~ 10	22 (27.50)	15 (25.00)	7 (29.17)		
> 10	18 (22.50)	13 (21.67)	5 (20.83)		

^*^P value: for comparison between elite and viremic controllers. N, number of patients

^a^ (n, %)

^b^ CD4 + T cell absolute counting (cell/μ L)

^c^^.^ Age,$mean$±SD, refers to the age at the follow-up in July 2022

### Survival Time and Survival Rates of LTNPs

**Fig. 2:**
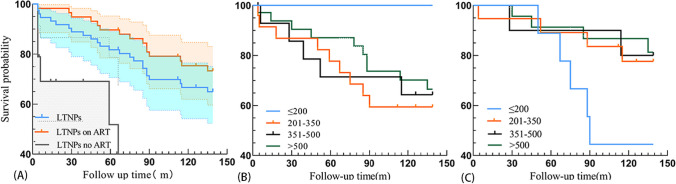
Comparison of LTNP survival curves from 2010 to 2022 (**A**: a comparison of survival curves among LTNPs (n = 80), LTNPs on ART (n = 60), and LTNPs no ART; **B**: A comparison of survival curves across different baseline CD4 count groups; **C**: A comparison of survival curves across different CD4 count groups at the start of ART)

In the 80 LTNPs, the effective observation time ranged from 4 to 139 months, with a median observation time of 126 months and an interquartile (IQR) of 46.25–139. The lowest survival rate within the first 8 years (until 2018) was 0.90. The cumulative survival rates at the 5th and 10th years of follow-up were 0.81 and 0.66, respectively (Table 2). The survival curves of LTNPs (n = 80), treated LTNPs (n = 60), and untreated LTNPs (n = 20) were significantly different (*χ*^*2*^ = 25.52, *P* < 0.001). No statistically significant differences were observed in survival curves when LTNPs were stratified by baseline CD4 + T cell counts (*χ*^*2*^ = 1.884, *P* = 0.597) or CD4 + T cell counts at different ART times (χ^2^ = 3.016, *P* = 0.083) (Fig. 2).

**Table 2:** Analysis of LTNPs survival rate from 2010 to 2022

	Follow-up time/year	Initial follow-up count	Censored data	Adjusted count	Number of deaths	In-period mortality rate	In-period survival rate	Cumulative survival rate	Cumulative survival rate Standard error
2010	0 ~	80	1	79.5	0	0.00	1.00	1.00	0.000
2011	1 ~	79	2	78.0	4	0.05	0.95	0.95	0.025
2012	2 ~	73	6	70.0	3	0.04	0.96	0.91	0.030
2013	3 ~	64	0	64.0	2	0.03	0.97	0.88	0.035
2014	4 ~	62	0	62.0	2	0.03	0.97	0.85	0.400
2015	5 ~	60	0	60.0	3	0.05	0.95	0.81	0.045
2016	6 ~	57	2	56.0	1	0.02	0.98	0.80	0.046
2017	7 ~	54	0	54.0	2	0.04	0.96	0.77	0.049
2018	8 ~	52	0	52.0	5	0.10	0.90	0.69	0.055
2019	9 ~	47	0	47.0	0	0.00	1.00	0.69	0.056
2020	10 ~	47	3	45.5	2	0.04	0.96	0.66	0.056
2021	11 ~	42	3	40.5	0	0.00	1.00	0.66	0.057
2022	12 ~	39	0	39.0	0	0.00	1.00	0.66	0.057

### Analysis of Factors Influencing Death in LTNPs

Log-rank tests noted that two factors (age at initial treatment and ART duration) affected the survival time among groups (P < 0.05). Additionally, three relevant factors (different modes of infection, baseline CD4 + T cell counts, CD4 + T cell counts at treatment initiation) were included in the multivariable Cox proportional hazards regression model, totaling five factors. The Cox model results indicated that individuals aged 31–40 years at treatment initiation had a higher risk of AIDS-related death than those aged over 50 years old at treatment initiation. Individuals with ART duration of less than 2 years exhibited a higher mortality risk, while LTNPs with ART durations of 3–10 years showed a reduced risk of mortality compared to untreated LTNPs.

### Demographic Characteristics of Three Different Groups of HIV-Infected Individuals

ART-treated LTNPs were matched in a 1:1 ratio with non-progressing HIV-infected individuals currently undergoing treatment, based on age, gender, and treatment duration (referred to as the “ART” group in the Table 3). Additionally, HIV-infected individuals who had never received ART were matched based on age and ethnicity (referred to as the "HIV" group in the subsequent text) (Table 4).

**Table 3:** Multivariate Cox regression analysis of LTNPs survival time from 2010 to 2022

	β	SE	*Waldχ*^*2*^	*P* value	*HR*	*95% CI*	
Baseline CD4 + T count^a,b^							
≤ 350			2.005	0.367			
351–500	− 0.606	0.836	0.526	0.468	0.545	0.106	2.805
> 500	− 0.915	0.647	1.997	0.158	0.401	0.113	1.424
Transmission route^a^							
IDU			0.972	0.808			
Spouse/fixed sex with positive	− 0.367	1.039	0.125	0.724	0.693	0.090	5.308
Non-marital, non-commercial heterosexual contact	0.490	0.837	0.343	0.558	1.632	0.316	8.424
Other	0.768	1.311	0.343	0.558	2.154	0.165	28.113
Age at time of start ART/year^a^							
Untreated			9.181	0.057			
≤ 30	2.300	1.349	2.909	0.088	9.977	0.710	140.262
31 ~ 40	2.693	1.069	6.348	0.012	14.775	1.819	120.033
41 ~ 50	1.836	1.085	2.864	0.091	6.269	0.748	52.549
> 50	5.951	2.245	7.027	0.008	384.300	4.717	31,308.778
CD4 + T cells at the start of ART^a,b^							
Untreated			1.834	0.766			
≤ 200	− 0.016	1.288	0.000	0.990	0.984	0.079	12.270
201–350	1.230	1.402	0.770	0.380	3.420	0.219	53.360
351–500	0.041	1.194	0.001	0.973	1.042	0.100	10.812
> 500	0.737	1.150	0.410	0.522	2.089	0.219	19.916
Duration of ART/year^a^							
Untreated			24.564	< 0.001	1		
≤ 2	0.942	0.903	1.090	0.296	2.566	0.437	15.049
3 ~ 5	− 3.095	1.277	5.873	0.015	0.045	0.04	0.553
6 ~ 10	− 4.189	1.306	10.292	0.001	0.015	0.01	0.196
> 10	− 15.819	194.005	0.007	0.935	0.000	0.000	1.853E + 158

^a^(n, %)

^b^CD4 + T cell absolute counting (cell/μL)

^c^Age,$mean$±SD, refers to the age at the follow-up in July 2022

**Table 4:** Demographic characteristics of different HIV infections in 2022

Variable	LTNP on ART [n (%)] (n = 23)	ART [n (%)] (n = 23)	PLWH [ (n)%] (n = 20)	χ^2^	*P* value
Age /years^b^					
≤ 40	1 (4.3)	3 (13.0)	4 (20.0)	5.596	0.627
41 ~ 50	16 (69.6)	14 (60.9)	12 (60.0)		
> 50	6 (26.1)	6 (26.1)	4 (20.0)		
Gender^a^					
Male	21 (91.3)	19 (82.6)	15 (75.0)	8.952	0.011
Female	2 (8.7)	4 (17.4)	5 (25.0)		
Age at HIV diagnosis /years^a^					
≤ 30	6 (26.1)	6 (26.1)	1 (5.0)	12.53	0.051
31 ~ 40	14 (60.9)	11 (47.8)	8 (40.0)		
41 ~ 50	3 (13.0)	5 (21.7)	7 (35.0)		
> 50	0 (0.0)	1 (4.3)	4 (20.0)		
Transmission route^a^					
IDU	16 (69.6)	10 (43.5)	3 (15.0)	19.836	0.003
Spouse/fixed sex with positive	2 (8.7)	8 (34.8)	5 (25.0)		
Non-marital, non-commercial heterosexual contact	5 (21.7)	3 (13.0)	11 (55.0)		
other	0 (0.0)	2 (8.7)	1 (5.0)		

^a^(n, %)

^b^Age, $mean$±SD, refers to the age at the follow-up in July 2022

### Immune Differences in Three Groups of HIV-Infected Individuals

The median and range of CD4 + T cell counts in LTNPs under treatment were 534 (146–1202), with a median and range of 3.96 (0.83–12.88) for natural killer (NK) cells (CD3-CD56 +), and 6.49 (3.90–17.64) for regulatory T (Treg) cells (CD4 + CD25 + CD127-). In the ART group, the median and range of CD4 + T cell counts were 546.5 (159–1376), NK cells was 1.22 (0.35–15.34), and Treg cells was 8.35 (3.34–13.70). For the HIV group, the median and range of CD4 + T cell counts were 403.5 (152–1439), NK cells was 0.63 (0.06–14.05), and Treg cells was 8.33 (3.44–17.42).

**Fig. 3:**
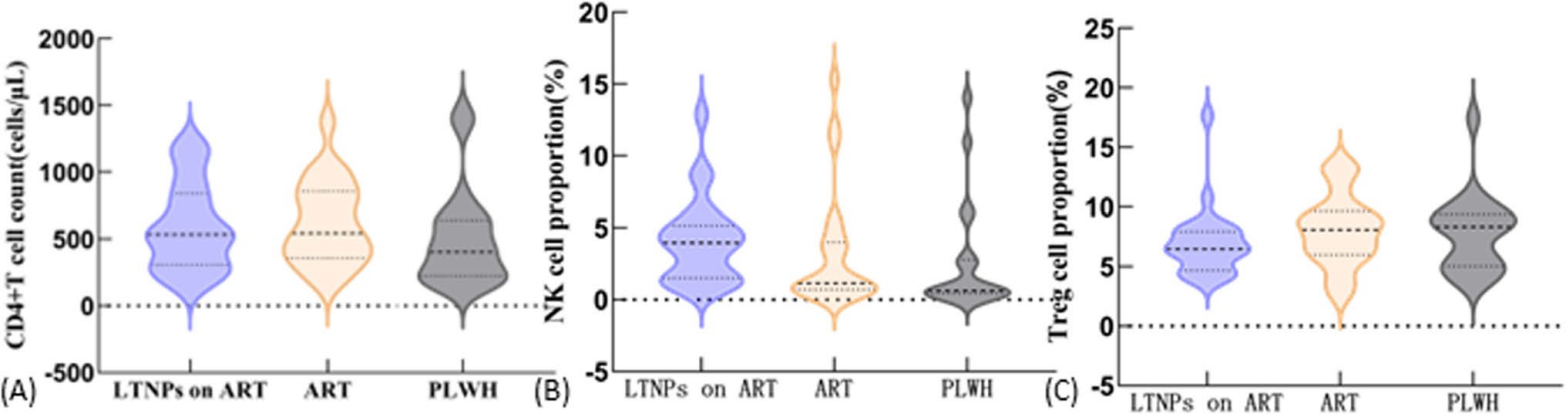
Immune differences among HIV-infected individuals in 2022: **A** Comparison of CD4 + T cell counts among the three groups; **B** Comparison of NK cell proportions among the three groups; **C** Comparison of Treg cell proportions among the three groups

Kruskal–Wallis test found a significant difference in NK cell proportions among the three groups (P = 0.005). However, there were no statistically significant differences in CD4 + T cell counts (P = 0.253) and Treg cell proportions (P = 0.237) among the three groups (both > 0.05) (Fig. 3).

**Fig. 4:**
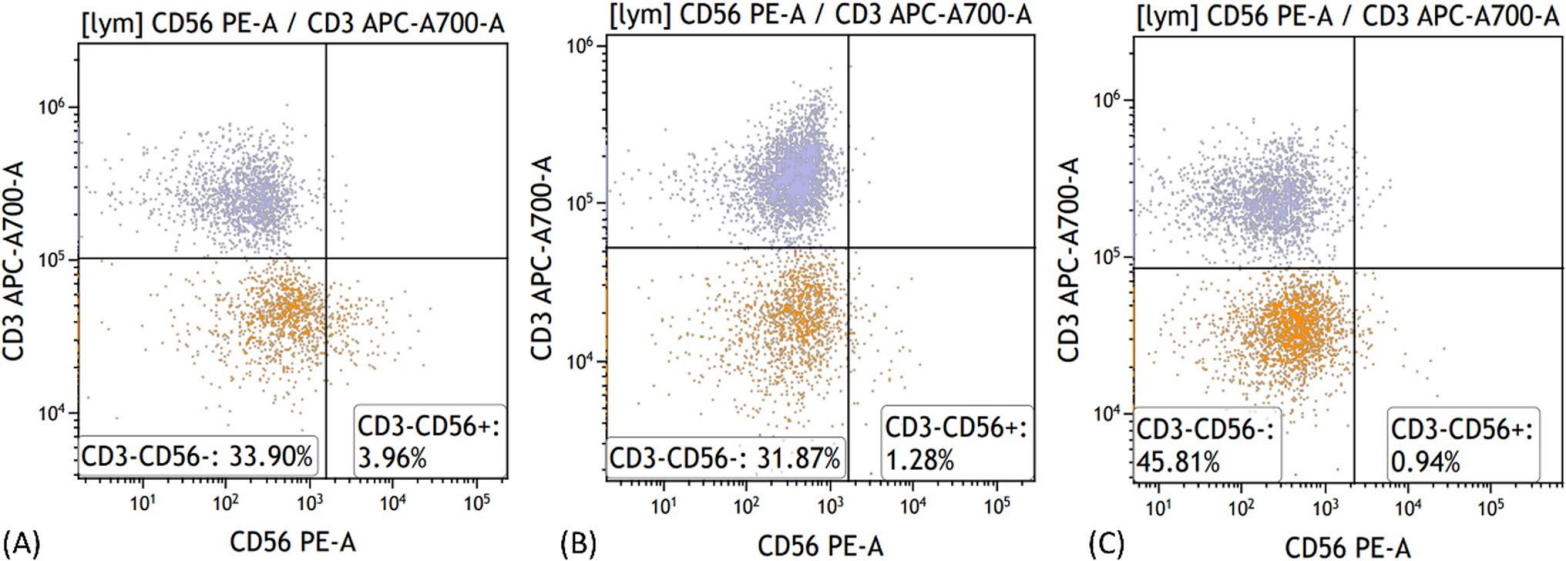
Comparative Analysis of NK Cell Proportions in Different HIV-infected Individuals in 2022 (**A**) LTNP on ART (**B**) ART (**C**) PLWH

Further pairwise comparisons of NK cell proportions among the three groups revealed that there was no statistically significant difference between the HIV group and the ART group (P = 0.183) and between the treated LTNPs group and the ART group (P = 0.052). However, there was a significant difference between the treated LTNPs group and the HIV group (P = 0.004) (Fig. 4).

## Discussion

As of 2021, there are 38.4 million people living with HIV/AIDS globally (33.9 million to 43.8 million) and 650,000 people (510,000 to 860,000) died from AIDS-related illnesses [6]. By the end of 2020, there were 49,357 surviving cases of HIV/AIDS in Xinjiang, ranking seventh in China. Without ART, most HIV-infected individuals develop AIDS within approximately 10 years [7]. With the continuous expansion of ART, HIV-infected individuals of different immune statuses are widely receiving treatment. ART has significantly reduced the incidence and mortality of HIV-infected individuals, substantially prolonging their survival [8].

Available evidence suggests that LTNPs may eventually lose viral control in the later stages of spontaneous viral control [9–11]. In 2020, Ezequiel expressed a preference for ART to ECs [5]. However, before immune dysfunction in LTNPs, it remains unknown whether policy-encouraged antiviral treatment can better help maintain viral control or extend the lifespan of this group. Interestingly, a recent study in Africa suggested that ART might potentially present more risks than advantages for some controllers [12]. The present study found significant differences in the survival of LTNPs receiving ART, mainly due to the small sample size of the untreated group. Therefore, this finding should not be taken as a conclusion. Notably, this research discovered that long-term ART (over two years) before symptoms appear in LTNPs could relatively reduce the risk of death and extend their lifespan to some extent, in line with previous conclusions [8], [13]. Consistently, a Barcelona research team in 2016, after six-year cohort observation, noted that ECs lost viral control at an average of 6.2 years, with nearly half of the patients showing CD4 progression, mainly due to follow-up duration, nadir CD4 + T cells, low-level viremia, and sexual risk [9]. Additionally, this study found that treatment initiation at a younger age might be associated with a lower risk of death, usually due to the accelerated aging associated with HIV infection [14], [15]. However, whether discontinuation of treatment causes LTNPs to become HIV post-treatment controllers [16] or rapid disease progression remains unknown and requires further research.

Previous studies have indicated that ART may directly and indirectly impact host immune function [17], [18]. There is also contentious discussion about the consequences of different ART regimens on immune activation and inflammation [19]. This study, through survival analysis, suggested that long-term ART may be a protective factor for LTNPs since it prolonged their lifespan to a certain extent. Furthermore, we found significant differences in NK cell proportions among LTNPs receiving ART, non-progressing HIV-infected individuals under treatment, and untreated HIV-infected individuals. NK cells are crucial antiviral effectors of the innate immune system [20]. They recognize stress, transformation, or infection signals and immediately exert effector functions. Reports have indicated that the regulatory function of NK cells is disrupted during HIV infection, and the activation state of NK cells is associated with disease progression [21, 22]. Additionally, NK cell activation is increased during chronic infection, even after virological suppression with ART [22, 23]. This study revealed a greater proportion of NK cells in LTNPs under ART than in other HIV-infected individuals, suggesting that ART does not compromise the inherent immune advantage of LTNPs.

This study also had limitations. Because of the unique nature of the study population, the sample size of this study was small. During the follow-up period, there was a significant loss of follow-up due to relocation and refusal of follow-up, which also caused instability in the COX regression model. Furthermore, 24 of the 80 LTNPs, died from causes that could not be definitively attributed to AIDS-related deaths. All reported deaths were recorded as follow-up outcomes. Additionally, most previous studies on disease progression in controllers have focused on ECs (defined as patients with two consecutive undetectable VLs without ART for at least one year), with minimal attention given to survival analysis of LTNPs receiving ART. Given that LTNPs and ECs both fall under the category of HIV controllers, they are compared in the discussion of risk factors in this study.

## Conclusion

ART for LTNPs does not cause NK cell dysfunction, and ART for over two years can prolong their survival to some extent. As an important group in the study of functional cures for HIV, LTNPs receiving ART are crucial for epidemiological and immunological studies. This study has important implications for the advancement of ART and the promotion of functional cures for AIDS. The life expectancy and quality of life of this group deserve ongoing attention.

## Supplementary Information

Below is the link to the electronic supplementary material.

Supplementary Material 1.

## Data Availability

The datasets generated or analyzed during this study are available from the corresponding author on reasonable request.
