# Supplementary material for: Survival Analysis and Immune Differences of HIV Long-Term Non-progressors in Xinjiang China: A 12-Year Prospective Cohort Observation
Source: AIDS Behav. 2024 Jun 13;28(9):3151–60. doi: 10.1007/s10461-024-04396-x (PMC11390762; doi:10.1007/s10461-024-04396-x)
Supplement: Supplementary file 1 — Supplementary Material 1. [file 10461_2024_4396_MOESM1_ESM.docx]

**[Supplementary material](https://www.ncbi.nlm.nih.gov/pmc/articles/PMC4863069/#SD1)**

**Table S1.** Basic Characteristics of LTNPs Alive in 2022 (Analyzed by Treatment Status)

| Variable | LTNP on ART (N=47) | LTNP no ART （N=9) | *χ²/t* | *P value** |
| --- | --- | --- | --- | --- |
| Gender ^a^ |  |  |  | 0.624^d^ |
| Male | 8(95.00) | 45(100.00) |  |  |
| Female | 1(5.00) | 2(0.00) |  |  |
| Age at HIV diagnosis /years ^b^ | 28.00(24.00-30.75) | 27.63±3.82 | 183.00 | 0.845 |
| transmission route ^a^ |  |  | 11.781 | 0.004 |
| IDU | 38(80.85) | 4(44.44) |  |  |
| Spouse/fixed sex with positive | 5(10.64) | 0(0.00) |  |  |
| Non-marital, non-commercial heterosexual contact | 3(6.38) | 4(44.44) |  |  |
| other | 1(2.13) | 1(0.11) |  |  |
| baseline CD4+T count ^a c^ | 549.00(473.25-685.75) | 609.50(473.25-685.75) | 185.50 | 0.881 |
| >10 | 13(21.67) | 5(20.83) |  |  |

*P value: for comparison between elite and viremic controllers. N, number of patients.

Gender、transmission route: Fisher's exact test; Age at HIV diagnosis /years: Wilcoxon Mann-Whitney test;

^a^ (n, %).

^b^ Median(*P*_25_-*P*_75_)

^c.^ CD4+T cell absolute counting (cell/μ L).
